# Supplementary material for: Significance of Lauren Classification in Patients Undergoing Neoadjuvant/Perioperative Chemotherapy for Locally Advanced Gastric or Gastroesophageal Junction Cancers—Analysis from a Large Single Center Cohort in Germany
Source: Cancers (Basel). 2021 Jan 14;13(2):290. doi: 10.3390/cancers13020290 (PMC7830383; doi:10.3390/cancers13020290)
Supplement: Supplementary file 1 [file cancers-13-00290-s001.pdf]

cancers MDPI

Supplementary Materials

Significance of Lauren Classification in Patients Undergoing Neoadjuvant/Perioperative Chemotherapy for Locally Advanced Gastric or Gastroesophageal Junction Cancers – Analysis from a Large Single Center Cohort in Germany

Rebekka Schirren, Alexander Novotny, Christian Oesterlin, Julia Slotta-Huspenina, Helmut Friess and Daniel Reim

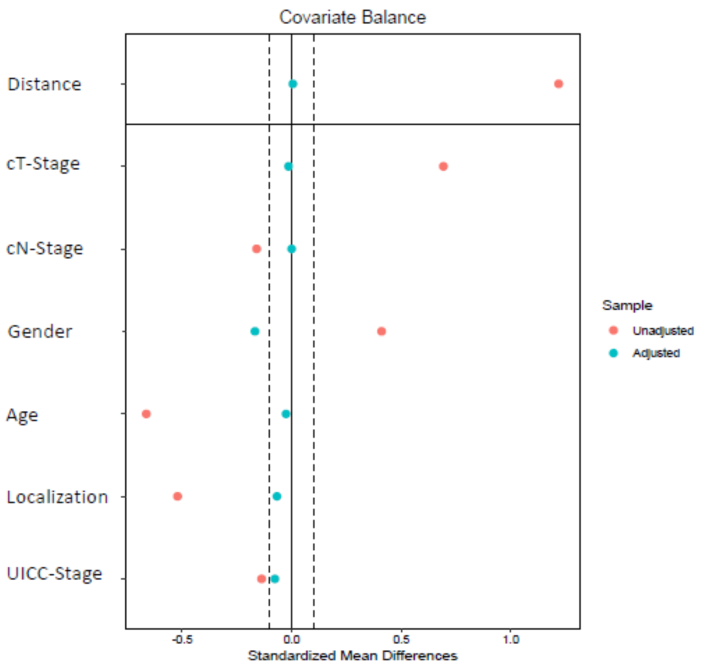

Figure S1: Love plot for propensity-score matching of confounding variables within the respective Lauren type subgroups.

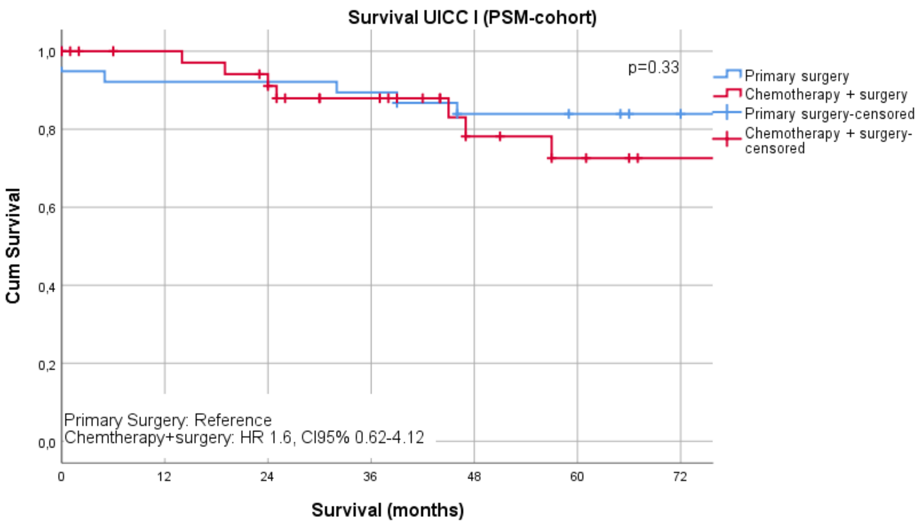

Figure S2. Kaplan Meier curve for OS in UICC stage I of PSM-cohort.

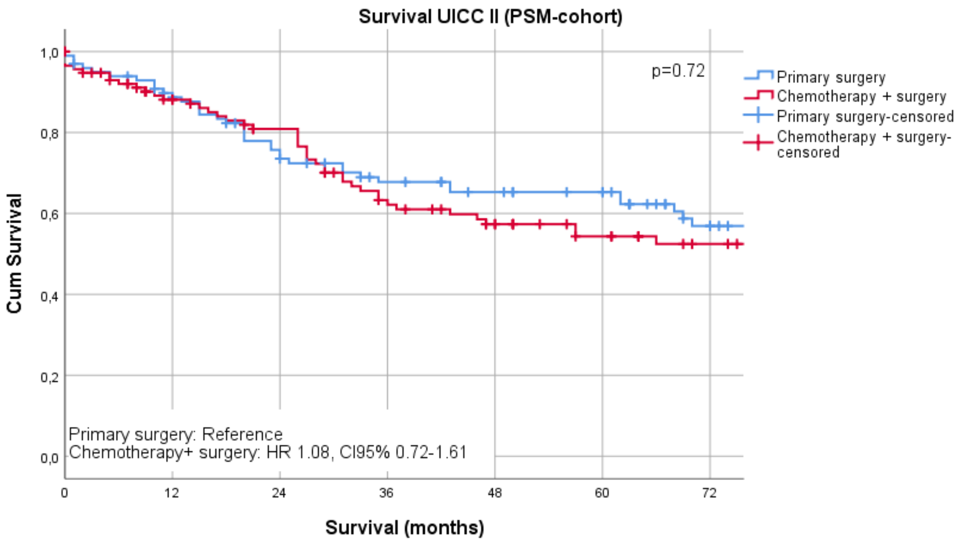

Figure S3. Kaplan Meier curve for OS in UICC stage II of PSM-cohort.

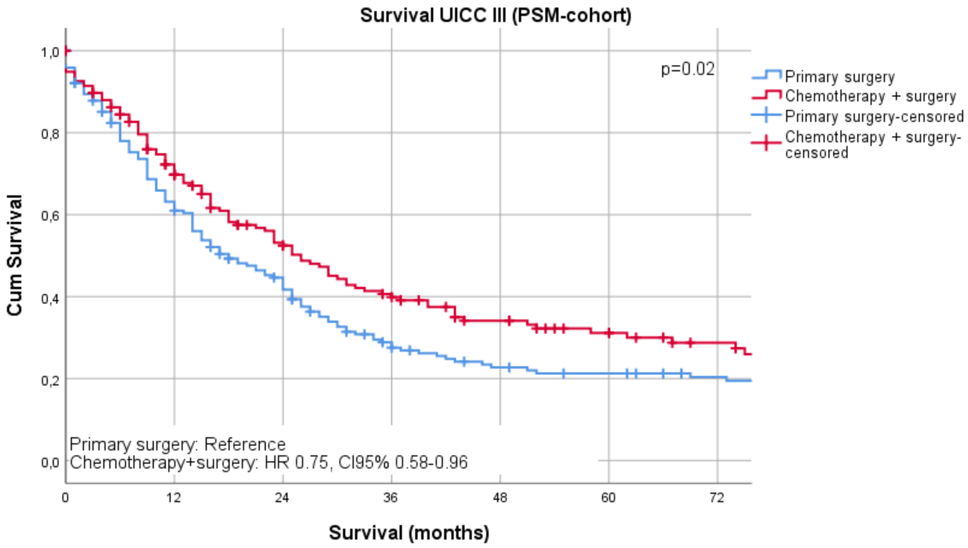

Figure S4. Kaplan Meier curve for OS in UICC stage III of PSM-cohort.

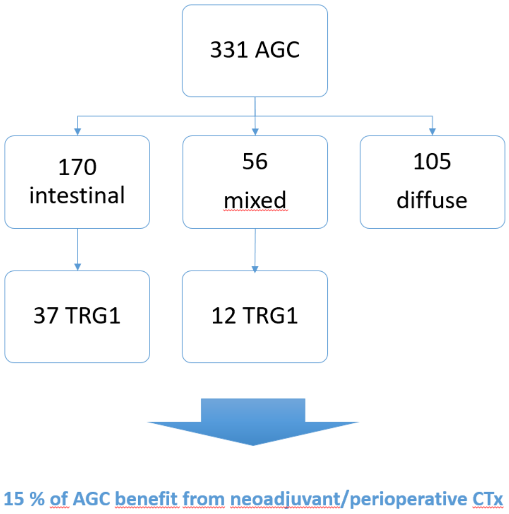

Figure S5. From 331 patients only 49 (15%) revealed histopathologic response and thus survival benefit.
